# Recent Northward Expansion of a Passerine Bird Species, Brownish-Flanked Bush Warbler (*Horornis fortipes*)

**DOI:** 10.3390/ani13233633

**Published:** 2023-11-23

**Authors:** Qianyi Zhang, Per Alström, Canwei Xia

**Affiliations:** 1 Ministry of Education Key Laboratory for Biodiversity and Ecological Engineering, College of Life Sciences, Beijing Normal University, Beijing 100875, China; zhangqianyi@mail.bnu.edu.cn; 2 Animal Ecology, Department of Ecology and Genetics, Evolutionary Biology Centre, Uppsala University, Norbyvägen 18 D, SE-752 36 Uppsala, Sweden; per.alstrom@ebc.uu.se

**Keywords:** *Horornis fortipes*, Cettiidae, range expansion, avian distributions, residence status, climate change

## Abstract

**Simple Summary:**

The distributions of many birds show a northward expansion in response to climate change. Brownish-flanked Bush Warbler (*Horornis fortipes*) is such a species, whose distribution expanded from 35° N to 40° N during the past decade. In this study, we documented the distribution of the newly established populations of this species and found evidence that it has bred at at least six sites north of its traditional range. Based on acoustic evidence, we inferred the source and expansion route of the individuals in Beijing and surrounding areas. This study is a case study of the dynamics of a bird species in the early stages of its northward expansion.

**Abstract:**

Northward expansions of bird distributions have been commonly observed in the Northern Hemisphere, likely as a result of climate change. The causes and ecological impacts of such range shifts have received extensive attention, but studies on the process of range shifts are still relatively scarce. The Brownish-flanked Bush Warbler (*Horornis fortipes*) has expanded northward from 35° N to 40° N during the past decade. In this study, we collated 77 records of the species beyond its traditional distribution during the past ten years from citizen science data. Most of the new records were from northeast of its traditional distribution, including the North China Plain, Taihang Mountains, and Taishan Mountain, and a few records from the northern margin of the Qinling Mountains and Qinghai–Tibet Plateau. We concluded that the Brownish-flanked Bush Warbler has bred in this new area in at least six sites. The newly established populations are assumed to belong to the subspecies *H. f. davidianus*, which can be divided into eastern and western dialect groups based on differences in songs. Song recordings from 10 males from Beijing and its adjacent areas were collected. Bayesian analysis based on the acoustic traits indicated that these males were most likely from the western dialect area, with a posterior probability of 99.975%. Combining topographical data with the habitat preference of the species, we inferred that these individuals spread northeastward from the Qinling Mountains to Taihang Mountains, and further along the Yanshan Mountains. This study is a case study of the distribution expansion of a bird species, which reflects the dynamics of a species in the early stage of its northward expansion.

## 1. Introduction

The global natural environment is undergoing dramatic changes due to climate change and the transformation of land use [1,2], which have effects on the phenology and physiology of organisms and lead to the distributional range shifts of many species [3,4,5]. Birds are sensitive to environmental change [6], and their distributions are affected by a combination of climatic conditions [7], habitat characteristics [8], human activities [9], and specific dispersal capacities [10]. Northward expansion of range margins has been commonly observed in the Northern Hemisphere [11,12,13]. Population densities of birds are also moving towards the poles, causing notable alterations in bird community compositions [14]. Range expansions of birds may have important ecological implications; for example, the Cattle Egret (*Bubulcus ibis*), which has spread from Africa to the Americas since the late 1800s, has experienced rapid expansion and population growth, and is competing with native herons for nesting sites [15]. The European Starling (*Sturnus vulgaris*), originally native to Europe and North Africa, was introduced to the Americas in the late 19th century, where it rapidly established colonies [16]. The species causes considerable damage to local agriculture in Italy [17], and has been found to pose a disease risk to livestock in the U.S. [18]. Studies on the island of Hawaii have shown that exotic birds alter the plant seed dispersal patterns [19]. Genetic introgression between the Ruddy Duck (*Oxyura jamaicensis*), which was introduced to Europe from North America, and White-headed Duck (*Oxyura leucocephala*) is posing a challenge to the conservation of White-headed Duck in Europe [20]. In addition, knowledge about species’ distributions under climate change is critical to biodiversity conservation and the design of management programs [21]. At present, the causes and ecological impacts of the spread of bird distributions have received extensive attention, but studies on the process of birds’ range shifts are still relatively scarce, and they have mostly focused on the establishment of introduced exotic birds [22,23,24], with less attention paid to the natural dispersal.

The family Cettiidae is a group of birds of the order Passeriformes, comprising about 31 species in seven genera (*Abroscopus*, *Phyllergates*, *Tickellia*, *Horornis*, *Tesia*, *Cettia*, and *Urosphena*), most of which are found in Eastern and Southern Asia, breeding in shrub and bamboo forests at medium-to-high altitudes [25,26]. Most of the species in this family are residents or short-distance migrants, except for the Asian Stubtail (*Urosphena squameiceps*) and the Manchurian Bush Warbler (*Horornis canturians*) [25,27]. The Brownish-flanked Bush Warbler (*Horornis fortipes*) is one of the resident birds of this family, which favors dense undergrowth, clearings with grasses, and grassy margins of mid-level forests, also edges of cultivation and bushy hillsides [28,29]. Brownish-flanked Bush Warbler contains four subspecies: *H. f. pallidus* in the Western Himalayas, *H. f. fortipes* in the Central and Eastern Himalayas, *H. f. robustipes* on the Island of Taiwan, and *H. f. davidianus* distributed from the southern parts of China to the northern part of Southeast Asia [25,28,29]. Genetic data suggested that the species contains three independent clades corresponding to *H. f. pallidus*, *H. f. fortipes*, and *H. f. davidianus* + *H. f. robustipes*; however, these lineages have hardly diverged in terms of morphometrics and only differ slightly in terms of plumage [30].

The northern boundary of the distribution of Brownish-flanked Bush Warbler was once considered to be the Qinling Mountains [31]. However, during the past 10 years, the species has been found in central Shanxi and Beijing, pushing its northern distribution limits from 35° N to 40° N [32]. However, field guides and fauna monographs published recently showed large differences in the northern part of the distribution of this species [32,33,34]. The first aim of this study is to collate the records of Brownish-flanked Bush Warbler beyond its traditional distribution area in order to clarify the actual distribution of its northward-expanding population. Understanding the residency of a species in an area is important to reveal its expansion process. The second aim is to focus on the new records of the Brownish-flanked Bush Warbler in Beijing and adjacent areas, which is a hotspot for birdwatching and where records have been increasingly collected since 2013, in order to further investigate its residence status. Although it has been suggested that the expanding population is of *H. f. davidianus* [32], we also try to determine the source of the individual birds in Beijing and its adjacent areas based on acoustic traits and speculate about their expansion route. Our study is a case study of the distribution expansion of a bird species, which reflects the dynamics of a species in the early stages of a northward expansion.

## 2. Materials and Methods

We collected records of Brownish-flanked Bush Warbler from citizen science data. Citizen science projects offer biological data that may fill information gaps left by other monitoring programs [35]. Birdreport (www.birdreport.cn, accessed on 31 July 2023) is one such project that has assembled more than 0.45 million reports from birdwatchers in China from 2014 and is an important source of data to monitor Chinese bird distribution dynamics [36]. The ebird (ebird.org) project provides a global online bird database that contains more than 8.29 million complete checklists [37]. We collected the records beyond the traditional distribution area of the Brownish-flanked Bush Warbler from the above two portals up to August 2023: birdreport.cn (accessed on 31 July 2023), ebird.org (accessed on 31 July 2023). The traditional distribution was defined by the distribution map in *Guide to the birds of China* [33], with some new distribution sites added according to *A checklist on the classification and distribution of the birds of China* (fourth edition) [32], which is similar to many fauna books and field guides of birds [28,38,39]. Additional records in Beijing since 2016 were added from the wildlife newsletter on Wild Beijing (birdingbeijing.com, accessed on 31 July 2023), which is a website dedicated to celebrating the wildlife in Beijing. The data from these projects suffer from problems such as large temporal and spatial spans and a lack of independence. Therefore, the records were revised with two principles. If a birdwatching tour lasted more than one day, the first day was recorded as the time when the species was observed. For multiple records from the same location on the same day, only the record with the earliest start time was retained.

Beijing is one of the earliest regions for birdwatching activities in China, and therefore, a large number of birdwatching data have been accumulated from there. For this reason, we focused on the records of Brownish-flanked Bush Warbler in Beijing and the surrounding 100 km. The distribution sites were classified into three categories: (i) presumed breeders, (ii) short-term visitors, and (iii) only sporadic records with unknown residency. As the nest of Brownish-flanked Bush Warbler is placed in dense undergrowth, breeding is rarely confirmed. The species breeds from May to August, with the incubation period of about 10 days and the brood period of about 14 days [29,39,40]. The Brownish-flanked Bush Warbler maintains a high degree of fidelity to its territory [41]. The species would be presumed to breed regularly at a site if there are records from that site during more than one breeding season, or there are records separated by more than one month during a breeding season. If there were several checklists from a certain site during a two-month period, but only one of these included Brownish-flanked Bush Warbler, the species would be considered to be a short-term visitor to the site. On the other hand, if the only birdwatching report in a two-month period from a certain locality included Brownish-flanked Bush Warbler, the residency was regarded as unknown there.

**Figure 1:**
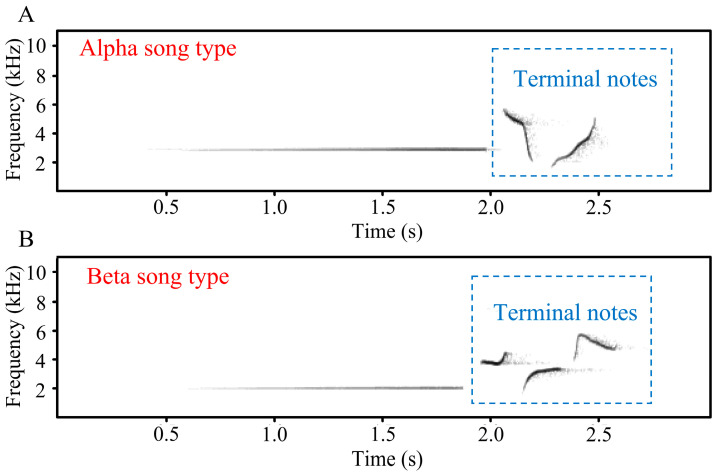
The song of Brownish-flanked Bush Warbler. Alpha song type has two terminal notes (**A**), while beta song type has three terminal notes (**B**).

We downloaded audio recordings pertaining to the birdwatching records. For records not including audio recordings, observers were contacted to request any sound recordings. The song of Brownish-flanked Bush Warbler consists of a whistle part followed by several terminal notes. According to the number of terminal notes, two commonly used song types can be identified, i.e., alpha song type (with two terminal notes) and beta song type (with three terminal notes) (Figure 1) [42]. For *H. f. davidianus*, the percentage of alpha song type among populations shows a clear geographic pattern, with nearly 50% alpha song type used in the western dialect zone and 76–100% alpha song type used in the eastern dialect zone, while there is no significant difference in other acoustic characteristics [28,40]. The boundary of the two dialect zones has been estimated at the line from Dabie Mountains to Luoxiao Mountains to Nanling Mountains [42].

Bayesian analysis was employed to infer the source of the individuals in the new distribution area (Beijing and its adjacent areas), based on the assumption that these individuals come from either the western or eastern dialect zone. First, two exclusive events were defined, i.e., A, from the western dialect zone, and A^C^, from the eastern dialect zone. The probability that a randomly selected song in the western dialect zone is of the alpha song type is 50%, while it is 88% in the eastern dialect zone, which is the mean of the alpha song type frequency in this dialect zone [42]. The probability of observed song type is equal to $\prod_{i=1}^{n}P(alpha\;song\;from\;individual\;i|A)$ or $\prod_{i=1}^{n}P(alpha\;song\;from\;individual\;i|A^{C}),$ where *n* is the number of recorded individuals. The posterior probability that the expanding population is from the western dialect zone is equal to $\frac{\prod_{i=1}^{n}P\left(alpha\;song\;from\;individual\;i|A\right)\cdot P\left(A\right)}{S}$, while the posterior probability that the expanding population is from the eastern dialect zone is equal to $\frac{\prod_{i=1}^{n}P(alpha\;song\;from\;individual\;i|A^{C})\cdot P(A^{C})}{S}$. S in the above expressions is the normalization factor and is equal to $\prod_{i=1}^{n}P(alpha\;song\;from\;individual\;i|A)\cdot P\left(A\right)+\prod_{i=1}^{n}P(alpha\;song\;from\;individual\;i|A^{C})\cdot P(A^{C})$. The prior probability for both $P\left(A\right)$ and $P\left(A^{C}\right)$ was set to 0.5.

## 3. Results

**Figure 2:**
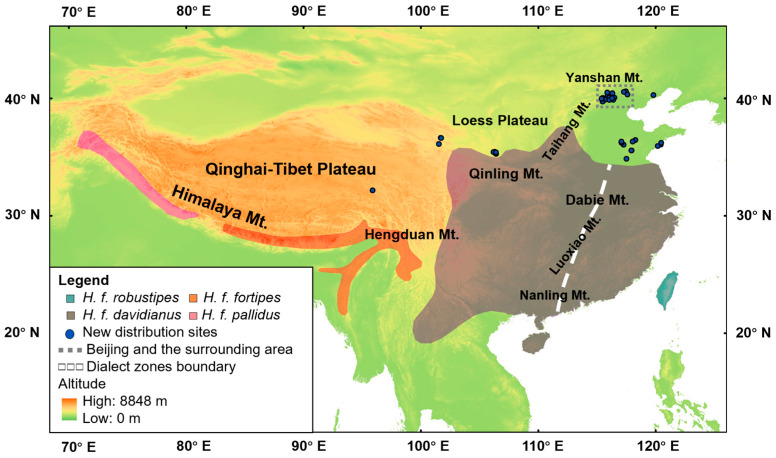
The updated distribution of Brownish-flanked Bush Warbler. The dotted square indicates the area shown in Figure 3.

In total, 77 records of Brownish-flanked Bush Warbler were collected from beyond the traditional distribution (Table S1). As shown in Figure 2, most of the new records were in the northeast of the traditional distribution, including the North China Plain, the Taihang Mountains, and Taishan Mountain (36°16′ N, 117°5′ E). There were a few records from the north margin of Qinling Mountains and Qinghai–Tibet Plateau. The updated northern limit of the distribution was in the Wuling Mountain (40°36′ N, 117°28′ E), Hebei Province, China.

**Figure 3:**
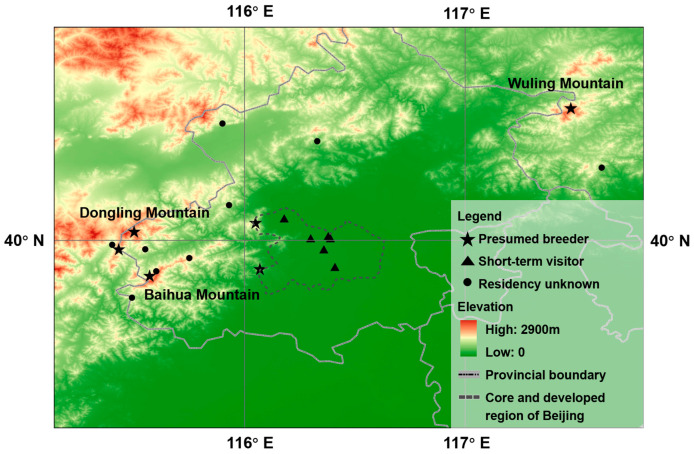
Records of Brownish-flanked Bush Warbler in Beijing and its adjacent areas within 100 km.

**Figure 4:**
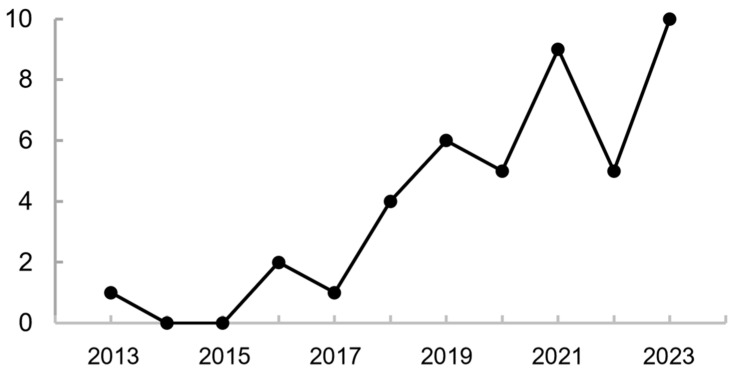
The increasing trend of records of Brownish-flanked Bush Warbler in Beijing and its adjacent area within 100 km from 2013 to 2023.

A total of 37 records were collected from Beijing and seven records from Hebei Province (Table 1, Figure 3). The records have been increasing since the species was first detected in Beijing in 2013 [43] and reached 10 in August 2023 (Figure 4). Specifically, Brownish-flanked Bush Warbler has been recorded during more than one breeding season at six sites and assumed to breed there. Short-term stays were noted at six other sites for which several birdwatching checklists were uploaded, but the species was recorded only once. The species was recorded only once at eight sites where the residency status was unknown and needs further observation.

It has been suggested, based on geographical proximity, that the northward-expanding population is *H. f. davidianus* [32], which could be further divided into two dialect groups [42]. Song recordings from 10 males at nine different locations from Beijing and Hebei were collected. The average number of strophes in these recordings was 3.9, with six recordings containing beta song type only and four recordings containing both alpha and beta song types. The results of the Bayesian analysis indicated that there was a 99.975% posterior probability that these males were from the western dialect area and less than 0.025% posterior probability that they were from the eastern dialect area.

## 4. Discussion

Many bird species are changing their distribution in response to global land use change and climate change [5,44,45]. The distributions of many Chinese birds show a current northward expansion, probably due to climate change [46,47,48]. The mechanisms of the expansion and the genetic and behavioral characteristics of the established populations have attracted much attention [49,50,51,52,53]. In this study, we documented the distribution of the expanding populations of Brownish-flanked Bush Warbler, which have moved northward from 35° N to 40° N over the past decade. We found evidence that the species has bred at six sites in Beijing and adjacent areas. We collected song recordings from 10 males from this area, and the acoustic analysis indicated that the males most likely originated from the western dialect area.

The northward-expanding populations were regarded to be *H. f. davidianus* because of the close distance to the traditional distribution of this subspecies [31,32,54]. In this study, most of the new distribution sites we collected were located in the northeast of the species’ traditional distribution range, where a wealth of recently published fauna studies is available. The absence of Brownish-flanked Bush Warbler in the earlier fauna records suggests a recent colonization of these areas [55,56,57]. In Qinling Mountains, the species was recorded earlier in 1987 [31], but no records of its presence were found in Liupan Mountain, located to the north of Qinling Mountains [58]. The observation in LiuPan Mountain (35°24′ N, 106°24′ E, Ningxia Hui Autonomous Region) could be attributed to the northward expansion of the Qinling population. As for the species’ distribution in the Qinghai–Tibet Plateau, documented sources indicated the presence of *H. f. fortipes* and *H. f. pallidus* in the south of the Himalayan Mountains [28,32,59]. In this study, the four records were collected in the northeastern margin of the Qinghai–Tibet Plateau, close to the Qinling population and likely to be *H. f. davidianus*. Notably, a record in Gaer Temple (32°9′ N, 95°53′ E, southern Qinghai Province) was collected that was closer to the northern limit of the distribution of *H. f. fortipes* (around 300 km) than to the western limit of the range of *H. f. davidianus* (around 500 km). However, this individual and the main population of *H. f. fortipes* are separated by the Himalayan Mountains that makes it more likely that it belonged to *H. f. davidianus*, but more evidence is needed to confirm this. Due to the vast expanse of the Qinghai–Tibet Plateau, the current observation effort may be insufficient. As a result, the species could have already expanded to other areas with limited birdwatching coverage.

In Beijing and adjacent areas, Brownish-flanked Bush Warbler has been observed for more than two consecutive years in spring and summer at six sites, suggesting breeding at those localities. Among the six sites, Baihua Mountain is popular for eco-tourism and is an important ecological barrier for Beijing city [60]. This was the highest site where the Brownish-flanked Bush Warbler was recorded, at over 1900 m, while Qianling Mountain (in Beijing) was the lowest elevation point where the species was recorded, at approximately 600 m. The altitude range is basically the same as the traditional distribution area (1200–1800 m) [28]. Their breeding habitats are characterized by dense undergrowth, bamboo clumps, and the middle strata of temperate forests, and also grassy edges and clearings thickets on hill sides, bush-covered hillsides and ravines, and scree slopes and occasionally in damper areas of valley bottoms [25]. The elevation similarity between the newly established populations and southern populations is likely a result of limited suitable breeding habitats at a low elevation in Beijing areas due to human activities and land-use changes. There were also eight sites around Beijing with only single records. These sites are located in the remote suburbs of the city, where few birdwatchers visit, and it is uncertain whether Brownish-flanked Bush Warbler breeds there or not. The species was also observed at six sites within Beijing city, including parks and campuses, which are typical urban garden habitats. These sites are easily accessible and are frequently visited by birdwatchers, as the Brownish-flanked Bush Warbler is uncommon in Beijing, and records of the species are of general interest to birdwatchers and researchers [61]. However, there were no more records after the first record at these sites within the same year, suggesting that Brownish-flanked Bush Warbler did not breed in urban garden habitats in Beijing. It is likely that the individuals recorded in the gardens of Beijing were in the process of dispersal. As non-native species should overcome a series of environmental obstacles to colonize a new environment, these continued dispersing individuals promote the establishment of new populations [62].

Based on the difference in the number of terminal notes, *H. f. davidianus* could be divided into two dialect groups [42]. Acoustic analyses showed that the newly established population around Beijing had a high degree of similarity to the western dialect area. The different dispersal ability between the eastern and western dialect groups suggested that they may be in the early stage of divergence, although no significant difference in genetics and morphology was found between the two dialect groups [30]. Furthermore, given the higher level of anthropogenic activities in the eastern region than in the western regions of the study area [63], more attention should be paid to the population dynamics of the eastern dialect group that has a lower dispersal ability. Considering that Brownish-flanked Bush Warbler inhabits hills and mountains [25,28,29], it is possible that the individuals in Beijing and adjacent area spread northeastward from the Qinling Mountains to Taihang Mountains (Xiaowutai Mountain in Hebei, Baihua Mountain and Dongling Mountain in Beijing), and further along the Yanshan Mountains to Wuling Mountain in Hebei and Liaoning Provinces. In recent years, there have been a number of new bird records in the high-altitude areas of Beijing in spring and summer, mainly of species found in central China. For example, the Grey-backed Shrike (*Lanius tephronotus*), found in Dongling Mountain in 2020, is mainly distributed in Northwestern and Central to Southwestern China [33,64,65]; the Ashy-throated Warbler (*Phylloscopus maculipennis*), recorded at an altitude of 2000 m in Baihua Mountain in 2019, is seen in Southern Tibet, Yunnan, and Sichuan in China [33,39,64]; and the Grey-winged Blackbird (*Turdus boulboul*), recorded in Dongling Mountain in 2016, is mainly distributed in Southwestern and Central China [33,64,66]. We can infer from records of these birds in Beijing that there may be a potential dispersal corridor from the Qinling Mountains via the Taihang Mountains to the Beijing area. As it has been shown that birds’ populations and distributions are sensitive to environmental changes, data from systematic monitoring and coordinated citizen science programmes of birds are critical for conservation management to fill information gaps [67,68,69]. As a result of the global climate change, more species from Central and Western China may be found in Beijing in the future. Regular and systematic surveys in Beijing’s high-altitude mountains are essential for effectively monitoring bird communities and ensuring the long-term biodiversity of the habitat.

The study of bird dispersal is essential for comprehending sink-source dynamics and colonization processes [70], which is also of critical importance for the conservation of species inhabiting fragmented habitats. The Brownish-flanked Bush Warbler is a year-round territorial bird and is usually regarded as a resident species within its traditional distribution area [41]. The study has revealed the expansion of its distribution range over the past decade. On the contrary, the Asian Stubtail and the Manchurian Bush Warbler, which belong to the same family Cettiidae, have long-distance migratory habits but have not been noted to expand their ranges northward [25,27]. This agrees with the previous findings that have suggested that there may not necessarily be a link between migratory strategy and dispersal ability in passerine birds [71]. Detailed information on the distribution of Brownish-flanked Bush Warbler around Beijing provides insights into the early establishment of a species during range expansion. Following the continuous growth of records of Brownish-flanked Bush Warbler, more detailed surveys will provide even more detailed insights into its population dynamics and ecological impacts.

## 5. Conclusions

In this study, we collated 77 records of Brownish-flanked Bush Warbler beyond the traditional distribution during the past ten years from citizen science data. Most of the new records were from the northeast of the traditional distribution, including the North China Plain, Taihang Mountains, and Taishan Mountain, and a few records were from the northern margin of the Qinling Mountains and Qinghai–Tibet Plateau. We concluded that the Brownish-flanked Bush Warbler has bred in at least six sites in Beijing and its surrounding areas. Song recordings from 10 males from this area were collected. Bayesian analysis based on the acoustic traits indicated that these males were most likely from the western dialect area. Combining topographical data with the habitat preference of the species, we inferred that these individuals spread northeastward from the Qinling Mountains to Taihang Mountains, and further along the Yanshan Mountains. In the future, more detailed surveys will provide insights into its population dynamics and ecological impacts.

## Figures and Tables

**Table 1:** Records of Brownish-flanked Bush Warbler in Beijing and its adjacent area.

Residency Type	Recording Site	Geographical Coordinates	Altitude (m)	Number of Independent Records
Presumed breeders	Baihua Mountain	39°50′ N, 115°34′ E	1900	9
	Dongling Mountain	40°2′ N, 115°30′ E	1596	5
	Miaofeng Mountain	40°5′ N, 116°3′ E	1124	4
	Qianling Mountain	39°52′ N, 116°4′ E	597	3
	Wuling Mountain	40°36′ N, 117°29′ E	1675	4
	Xiaolongmen	39°58′ N, 115°26′ E	1219	3
Short-term visitors	Olympic Forest North Park	40°1′ N, 116°23′ E	37	1
	Olympic Forest South Park	40°1′ N, 116°23′ E	40	1
	Beijing Normal University	39°58′ N, 116°22′ E	51	1
	Cuihu Wetland Park	40°6′ N, 116°11′ E	42	1
	The Temple of Heaven Park	39°53′ N, 116°25′ E	44	2
	Yuanmingyuan Park	40°1′ N, 116°18′ E	44	1
Residency unknown	Huangantuo	39°52′ N, 115°36′ E	1393	1
	Daan Mountain	39°55′ N, 115°45′ E	1488	1
	Erhuangtai	40°27′ N, 116°20′ E	396	1
	Laoyugou	40°10′ N, 115°56′ E	1234	1
	Puwa	39°44′ N, 115°30′ E	1073	1
	Xiaowutai Mountain	39°59′ N, 115°24′ E	970	1
	Kulong Mountain	39°58′ N, 115°33′ E	583	1
	Liuliping	40°20′ N, 117°37′ E	528	1
	Yudu Mountain	40°32′ N, 115°54′ E	887	1

## Data Availability

The distributional data in this study are presented in the supplementary material, and the song recordings are available on request from the corresponding authors.

## Supplementary Materials

The following supporting information can be downloaded at: https://www.mdpi.com/article/10.3390/ani13233633/s1, Table S1: New records of Brownish-flanked Bush Warbler (*Horornis fortipes*) beyond its traditional distribution during 2013 to 2023.
